# The impact of surgery on patients with Von Hippel-Lindau-associated tumors: an international patient survey

**DOI:** 10.1093/oncolo/oyaf206

**Published:** 2025-08-12

**Authors:** Murali Sundaram, Christian Atkinson, Charley Cooper, Gavin Taylor-Stokes, Joshua Mann, Othon Iliopoulos

**Affiliations:** Merck & Co. Inc., Rahway, NJ 07065, United States; Adelphi Real World, Bollington, SK10 5JB, United Kingdom; Adelphi Real World, Bollington, SK10 5JB, United Kingdom; Adelphi Real World, Bollington, SK10 5JB, United Kingdom; VHL Alliance, Boston, MA 02132, United States; Massachusetts General Hospital Center of Cancer Research and Harvard Medical School, Boston, MA 02114, United States

**Keywords:** Von Hippel-Lindau syndrome, surgery outcomes, tumor management, real-world

## Abstract

**Background:**

Von Hippel-Lindau syndrome (VHL) is a rare hereditary neoplastic disorder caused by mutations in the *VHL* gene. Treatment options for patients are limited to multiple surgeries dispersed between regular scans, watchful waiting, and treatments that preserve organ function.

**Methods:**

An international, cross-sectional survey comprising patients in the United States (USA), Canada (CA), the United Kingdom (UK), France (FR), and Germany (DE) was conducted. Patients were recruited via the VHL Alliance; data were collected between Dec 2021 and May 2022. For inclusion, patients must have renal cell carcinoma, pancreatic neuroendocrine tumors, and/or central nervous system hemangioblastoma.

**Results:**

In all, 220 patients (68.2% female, median age 40.0, median disease duration 15.8 years) in the USA (*n* = 108), CA (*n* = 37), the UK (*n* = 21), FR (*n* = 3), and DE (*n* = 51) completed the study. In this sample, *n* = 205 (93.2%) patients had experienced surgery; *n* = 171 (77.7%) had experienced multiple surgeries (median number of surgeries, 4.0); 166 (*n* = 75.5%) patients recorded data on their most recent surgery. Of these, patients reported that their most recent surgery worsened (scored 1-3) their fatigue (51.8%, *n* = 86), mental health (51.2%, *n* = 85), and ability to go about daily life (45.2%, *n* = 75). Approximately, 47.3% (*n* = 104) of patients selected reducing the number of surgeries as a top treatment goal, whereas 73% (*n* = 161) of patients indicated they would prefer to take a pill which would possibly delay the time until surgery.

**Conclusion:**

Surgery negatively impacts the lives of patients, leading to a worsening in their fatigue, mental health, and ability to go about daily life.

Implications for Practice The findings highlight the significant burden of repeated surgeries on patients with Von Hippel-Lindau disease, negatively impacting their fatigue, mental health, and daily functioning. Patients express a strong preference for treatments that reduce or delay the need for surgery, with nearly three-quarters indicating they would opt for a pill to achieve this. These insights underscore the urgent need for alternative therapeutic strategies that prioritize preserving the quality of life while managing tumor progression. Clinicians should consider patient preferences when discussing treatment plans, and further research into nonsurgical interventions is warranted to address these unmet needs.

## Introduction

Von Hippel-Lindau disease (VHL) is a rare hereditary neoplastic disorder caused by germline mutations in the *VHL* gene, leading to the development of tumors in several organs, including the central nervous system, pancreas, kidneys, and reproductive organs.^1^ Renal cell carcinoma (RCC) is the most frequent visceral manifestation and most frequent type of malignant tumor in VHL. The reported frequency of RCC in VHL is estimated at 31% worldwide (ranging from 13% to 62%).^2^ Central nervous system hemangioblastomas (CNS-Hb) are the most frequent tumor type affecting different parts of CNS (including cerebellum, spine, brainstem, cerebrum, and retina of the eye) and are estimated to affect up to 79% of patients with VHL worldwide.^2^ Pancreatic neuroendocrine tumors (pNET) are another manifestation associated with VHL. Approximately 11% of VHL patients worldwide will develop pNET (ranging from 2% to 17%).^2^^,^^3^

Historically, treatment options for VHL patients consisted mainly of multiple surgeries^4^ and/or radiotherapy for CNS-Hb. Surgical interventions are typically guided by factors such as tumor size, growth rate, and associated symptoms. For instance, RCCs are often resected before tumors exceed 3 cm in diameter to minimize the risk of malignant progression and preserve kidney function. Microsurgical resection is the preferred treatment for CNS hemangioblastomas, with stereotactic or craniospinal radiation utilized for patients who are not suitable candidates for surgery. Due to the unpredictable growth patterns of VHL-related hemangioblastomas, prophylactic radiation is not recommended, except for tumors that are inaccessible or cannot be safely removed surgically.^5^^,^^6^

These interventions were decided after a watchful waiting approach, whereby tumor growth is surveyed using serial imaging studies (mostly Magnetic Resonance Imaging [MRIs]). The challenge of repeated surgical and/or radiotherapy interventions is to prevent metastatic disease, while preserving organ function; to this end treatment approaches to VHL-related tumors involved radiofrequency ablation, cryotherapy, and partial nephrectomy for localized RCC, partial pancreatectomy for pNET tumors, and craniotomy or stereotactic radiosurgery for CNS tumors. However, organ function eventually decreases with multiple surgical procedures resulting in significant morbidity and mortality from surgery and/or radiation.^7^^,^^8^

Considering the long-term impact of VHL on quality of life, mental health, and other factors like productivity, it is worthwhile to examine how diseases with limited treatment options can lead to physical and psychosocial impairments, as well as the potential impact of an approved and available systemic therapy.^9^ It has been scientifically evaluated that the outlook on cancer-related imaging/scans already has an impact on the mental health of the patients. So called “scanxiety”, or the distress and/or anxiety occurring before, during, and after medical scans, describes the apprehension of getting poor results that is involved in the experience.^10^^,^^11^ Together with the prospect of VHL patients having to go for frequent imaging and subsequent surgeries, this may have a significant impact on patients’ quality of life, mental health, and overall ability to cope with VHL disease.

The introduction of systemic therapy with the hypoxia-inducible factor-2 alpha (HIF-2α) inhibitor belzutifan has offered a novel therapeutic option for patients with VHL. Belzutifan was approved by the Food and Drug Administration in 2021 for treating adult patients with VHL disease who require therapy for RCC, CNS hemangioblastomas, or pNET, and do not require immediate surgery.^12^ The LITESPARK-004 trial demonstrated promising results, with belzutifan showing a 49% objective response rate (ORR) in RCC lesions, a 44%-76% ORR in CNS hemangioblastomas, as well as stabilization or regression of pNET tumors.^13^^,^^14^ This availability of a nonsurgical option may significantly alter the perspective of VHL patients on disease management and overall quality of life.

The aim of this study was to evaluate the impact of VHL-related surgeries on patients’ lives, and whether patients would prefer alternative treatment that could potentially alleviate the need for surgery.

## Material and methods

### Study design

The VHL patient survey was an international, cross-sectional study comprising patients in the United States (USA), Canada (CA), the United Kingdom (UK), France (FR), and Germany (DE). Data were collected between December 2021 and May 2022. The study used the EQ-5D-5L (European Quality of Life, 5 Dimensions) to evaluate the impact of VHL on patients’ lives. The EQ-5D-5L is a pivotal tool in assessing self-reported health status and health-related quality of life since the 1990s. In the “5D-5L” setting, there are five questions with five possible answers each. The survey was designed to assess the mobility, the self-care ability, and possible complications when doing usual activities, including pain/discomfort and anxiety/depression. The protocol and supporting data collection materials were approved by the Western Institutional Review Board, USA. The study was conducted in accordance with the ethical principles that have their origin in the Declaration of Helsinki and that are consistent with Good Pharmacoepidemiology Practices and applicable laws and regulations of the countries where the study was conducted, as appropriate. Study materials may be provided upon reasonable request.

### Participants

For inclusion, patients were required to be 18 years old and must have been diagnosed with VHL-related RCC, pNET, and/or CNS-Hb. Patients were recruited online via a VHL advocacy group—the VHL Alliance. Patients were asked to complete one structured survey, designed to be completed in one sitting. A unique identifier was created for each patient to prevent the duplication of data. Once the database was prepared and finalized, all identifiers were removed and data were anonymized. Participation in the survey was voluntary. All participants provided written informed consent before participating in the study. Gender data were self-reported, and selected from a list of: male, female, intersex, prefer not to say.

### Procedures

The survey material was developed in English, translated into native languages, and delivered online. The completion of the survey took the participants approximately 30 minutes. Data collected included demographics, symptoms, experiences with surgery, treatments, and the EQ-5D-5L. In order to assess patient preference, the following scenarios and options were presented in the questionnaire: “Imagine that you have not had VHL-related surgery in the past. You now have VHL-related cancer, but you are not yet eligible to have surgery on that tumor. You have the following options:”

The first scenario and options were as follows:

1. **“Watch and wait”:** You will continue with regular doctor visits and monitor changes to your VHL-related tumor over time. At some point, if the tumor and/or tumor-associated cyst grows, you will need to have surgery.
2. **“Take a pill”:** You will continue with regular doctor visits and monitor changes to your VHL-related tumor over time. At some point, if the tumor grows, you will need to have surgery. You will also take a pill once a day. This pill may have some side effects but could slow the growth of your VHL-related tumor and possibly delay the time to surgery.

The second scenario and options were as follows:

1. **“Watch and wait”:** You will continue with regular doctor visits and monitor changes to your VHL-related tumor over time. At some point, if the tumor and/or tumor-associated cyst grows, you will need to have surgery.
2. **“Take a pill”:** You will continue with regular doctor visits and monitor changes to your VHL-related tumor over time. At some point, if the tumor and/or tumor-associated cyst grows, you will need to have surgery. You will also take a pill once a day. This pill may have some side effects but could slow the growth of your VHL-related tumor and possibly delay the time until you need to have surgery. **In addition, the pill may also slow the growth of other VHL-related tumors and/or tumor-associated cysts**.

### Minimization of bias

The following steps were taken towards minimizing bias: where applicable, neutral phrasing of questions was used to avoid biasing responses. In addition, response options were exhaustive, including “other” and “don’t know” response options, to avoid respondents having to enter false information in order to progress through the survey. Furthermore, recruitment of a relatively large patient sample with minimal inclusion criteria from a recognized VHL patient support association ensured a diverse representation of perspectives and experiences.

### Statistical analysis

All the analyses were descriptive, and no formal hypothesis was tested.

## Results

In total, 220 patients from around the world completed the study, including patients in USA (*n* = 108, 49.1%), Germany (*n* = 51, 23.2%), Canada (*n* = 37, 16.8%), the UK (*n* = 21, 9.5%), and France (*n* = 3, 1.4%). The patients were 68.2% (*n* = 150) female, had a median age 40.0 years, and a median disease duration of 15.8 years. About 88.6% (*n* = 195) of the patients were White, 4.1% (*n* = 9) were Hispanic/Latino, 0.9% (*n* = 2) were Mixed Race, 0.9% (*n* = 2) were from the Asian-Indian subcontinent, 0.9% (*n* = 2) were South-East Asian, 0.5% (*n* = 1) were Chinese, 0.5% (*n* = 1) were Japanese, 0.5% (*n* = 1) were Korean, 0.5% (*n* = 1) were Asian (other), and 2.3% (*n* = 5) were Other.

When asked about VHL manifestations, 146 (66.4%) patients reported RCC, 122 (55.5%) patients reported pNETs, and 190 (86.4%) patients reported CNS-Hb. The top symptoms experienced by VHL patients were numbness (*n* = 59, 26.8%), dizziness (*n* = 56, 25.5%), and fatigue/low energy (*n* = 54, 24.5%). The top comorbid condition experienced alongside VHL were anxiety (*n* = 78, 35.5%), depression (*n* = 63, 28.6%), and high blood pressure (*n* = 61, 27.7%). Full demographic information and clinical characteristics are reported in Table 1.

**Table 1.** Demographics and clinical characteristics.

	Overall	RCC	pNET	CNS-Hb
*n*, (%)	220 (100)	146 (66.4)	122 (55.5)	190 (86.4)
Patient age, median (interquartile range)	40.0 (30.2-53.0)	41.0 (30.0-53.2)	41.0 (31.0-54.0)	41.0 (31.0-54.0)
Patient sex assigned at birth, *n* (%)				
Female	150 (68.2)	96 (65.8)	81 (66.4)	126 (66.3)
Male	70 (31.8)	50 (34.2)	41 (33.6)	64 (33.7)
Patient ethnicity, *n* (%)				
White	195 (88.6)	128 (87.7)	108 (88.5)	171 (90)
Hispanic/Latino	9 (4.1)	7 (4.8)	3 (2.5)	7 (3.7)
Asian Indian subcontinent	2 (0.9)	1 (0.7)	1 (0.8)	1 (0.5)
Korean	2 (0.9)	1 (0.7)	1 (0.8)	2 (1.1)
Mixed race	2 (0.9)	1 (0.7)	1 (0.8)	2 (1.1)
South-East Asian	2 (0.9)	2 (1.4)	2 (1.6)	0 (0)
Asian (other)	1 (0.5)	0 (0)	1 (0.8)	1 (0.5)
Chinese	1 (0.5)	1 (0.7)	1 (0.8)	1 (0.5)
Japanese	1 (0.5)	1 (0.7)	1 (0.8)	1 (0.5)
Other	5 (2.3)	4 (2.7)	3 (2.5)	4 (2.1)
Days since diagnosis of VHL, median (interquartile range)^a^	5751.0 (2932.0-9155.0)	5992.0 (3/024.5-9423.0)	5463.0 (2188.0-9783.0)	5812.5 (2944.0-8974.8)
Tumors diagnosed with, *n* (%)				
RCC + pNET + CNS-Hb	77 (35)	77 (52.7)	77 (63.1)	77 (40.5)
RCC + CNS-Hb	43 (19.5)	43 (29.5)	0 (0)	43 (22.6)
CNS-Hb	39 (17.7)	0 (0)	0 (0)	39 (20.5)
pNET + CNS-Hb	31 (14.1)	0 (0)	31 (25.4)	31 (16.3)
RCC	16 (7.3)	16 (11)	0 (0)	0 (0)
RCC + pNET	10 (4.5)	10 (6.8)	10 (8.2)	0 (0)
pNET	4 (1.8)	0 (0)	4 (3.3)	0 (0)
Symptoms/signs currently experiencing, *n* (%)				
Kidney cysts	82 (37.3)	82 (56.2)	46 (37.7)	63 (33.2)
Numbness	59 (26.8)	30 (20.5)	33 (27)	59 (31.1)
Dizziness	56 (25.5)	24 (16.4)	29 (23.8)	56 (29.5)
Fatigue/low energy	54 (24.5)	54 (37)	28 (23)	44 (23.2)
Headaches	46 (20.9)	46 (31.5)	29 (23.8)	38 (20)
High blood pressure	45 (20.5)	45 (30.8)	21 (17.2)	37 (19.5)
Pain in your lower back	34 (15.5)	34 (23.3)	19 (15.6)	26 (13.7)
Weakness in the arms	33 (15)	16 (11)	16 (13.1)	33 (17.4)
Incoordination	32 (14.5)	18 (12.3)	15 (12.3)	32 (16.8)
Vertigo	32 (14.5)	14 (9.6)	18 (14.8)	32 (16.8)
Weakness in the legs	30 (13.6)	15 (10.3)	15 (12.3)	30 (15.8)
Difficult concentrating	30 (13.6)	30 (20.5)	19 (15.6)	26 (13.7)
Pain/aches in your muscles	29 (13.2)	29 (19.9)	16 (13.1)	22 (11.6)
Hearing loss	28 (12.7)	19 (13)	15 (12.3)	28 (14.7)
Tinnitus	26 (11.8)	12 (8.2)	16 (13.1)	26 (13.7)
Bloating	23 (10.5)	9 (6.2)	23 (18.9)	21 (11.1)
Memory loss	23 (10.5)	23 (15.8)	16 (13.1)	19 (10)
Abdominal pain	21 (9.5)	9 (6.2)	21 (17.2)	19 (10)
Blurred vision	20 (9.1)	20 (13.7)	13 (10.7)	17 (8.9)
Night sweats	20 (9.1)	20 (13.7)	8 (6.6)	14 (7.4)
Pain in your abdomen/stomach	20 (9.1)	20 (13.7)	14 (11.5)	15 (7.9)
Weight gain	20 (9.1)	20 (13.7)	9 (7.4)	15 (7.9)
Cramping	17 (7.7)	5 (3.4)	17 (13.9)	16 (8.4)
Nausea (feeling sick)	17 (7.7)	17 (11.6)	10 (8.2)	12 (6.3)
Pain in your side (flank)	17 (7.7)	17 (11.6)	9 (7.4)	14 (7.4)
Weight loss	15 (6.8)	10 (6.8)	11 (9.0)	11 (5.8)
Diarrhea	12 (5.5)	12 (8.2)	9 (7.4)	10 (5.3)
Loss of appetite	12 (5.5)	12 (8.2)	7 (5.7)	7 (3.7)
Shortness of breath	12 (5.5)	12 (8.2)	8 (6.6)	9 (4.7)
Fatty stool	11 (5)	5 (3.4)	11 (9)	9 (4.7)
Swelling of your ankles	10 (4.5)	10 (6.8)	4 (3.3)	9 (4.7)
Incontinence	8 (3.6)	5 (3.4)	3 (2.5)	8 (4.2)
Pain in your bones	8 (3.6)	8 (5.5)	4 (3.3)	6 (3.2)
Skin rash	6 (2.7)	6 (4.1)	2 (1.6)	5 (2.6)
Vomiting (throwing up)	6 (2.7)	6 (4.1)	2 (1.6)	3 (1.6)
A mass/lump on your back	5 (2.3)	5 (3.4)	4 (3.3)	2 (1.1)
Swollen lymph nodes	5 (2.3)	5 (3.4)	1 (0.8)	3 (1.6)
A mass/lump on your side (flank)	3 (1.4)	3 (2.1)	1 (0.8)	1 (0.5)
Blood in urine	3 (1.4)	3 (2.1)	1 (0.8)	1 (0.5)
Fever	1 (0.5)	1 (0.7)	0 (0)	0 (0)
Jaundice	1 (0.5)	0 (0)	1 (0.8)	1 (0.5)
Other	18 (8.2)	9 (6.2)	10 (8.2)	18 (9.5)
None	29 (13.2)	14 (9.6)	14 (11.5)	27 (14.2)
Comorbid conditions currently experiencing, *n* (%)				
Anxiety	78 (35.5)	45 (30.8)	43 (35.2)	70 (36.8)
Depression	63 (28.6)	40 (27.4)	32 (26.2)	57 (30)
High blood pressure	61 (27.7)	46 (31.5)	30 (24.6)	52 (27.4)
None	47 (21.4)	30 (20.5)	26 (21.3)	39 (20.5)
High cholesterol	41 (18.6)	26 (17.8)	20 (16.4)	35 (18.4)
Chronic pain	40 (18.2)	23 (15.8)	23 (18.9)	35 (18.4)
Diabetes	35 (15.9)	25 (17.1)	24 (19.7)	27 (14.2)
Hearing loss	33 (15)	22 (15.1)	22 (18)	31 (16.3)
Other (please specify)	29 (13.2)	21 (14.4)	12 (9.8)	26 (13.7)
Low blood pressure	17 (7.7)	10 (6.8)	11 (9)	16 (8.4)
Asthma or chronic obstructive pulmonary disease	15 (6.8)	9 (6.2)	10 (8.2)	12 (6.3)
Other cancer	14 (6.4)	12 (8.2)	10 (8.2)	10 (5.3)
Motor loss	14 (6.4)	4 (2.7)	5 (4.1)	14 (7.4)
Chronic kidney disease (long term reduced kidney function, not RCC)	13 (5.9)	9 (6.2)	7 (5.7)	8 (4.2)
Heart disease	12 (5.5)	12 (8.2)	8 (6.6)	10 (5.3)
Ataxia	12 (5.5)	7 (4.8)	4 (3.3)	12 (6.3)
Liver disease	11 (5)	8 (5.5)	8 (6.6)	9 (4.7)
Hypoglycemic syndrome	2 (0.9)	0 (0)	0 (0)	2 (1.1)
Type of tumor, *n* (%)				
RCC	146 (66.4)	146 (100)	87 (71.3)	120 (63.2)
pNET	122 (55.5)	87 (59.6)	122 (100)	108 (56.8)
CNS-Hb	190 (86.4)	120 (82.2)	108 (88.5)	190 (100)

a *n* = 21 patients answered “Don’t know” when asked for the time since diagnosis of VHL.

Abbreviations: VHL, Von Hippel-Lindau syndrome; RCC, renal cell carcinoma; pNET, pancreatic neuroendocrine tumors; CNS-Hb, central nervous system hemangioblastomas.

**Figure 1.**
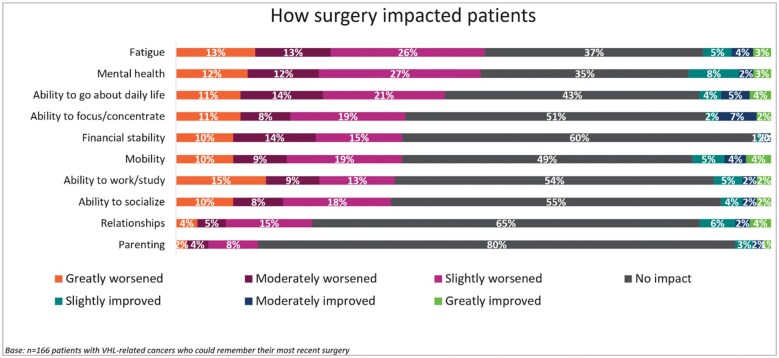
Overall impact of surgery on patients with VHL disease. VHL, Von Hippel-Lindau disease, *n* = 39 patients could not remember their most recent surgery.

**Figure 2.**
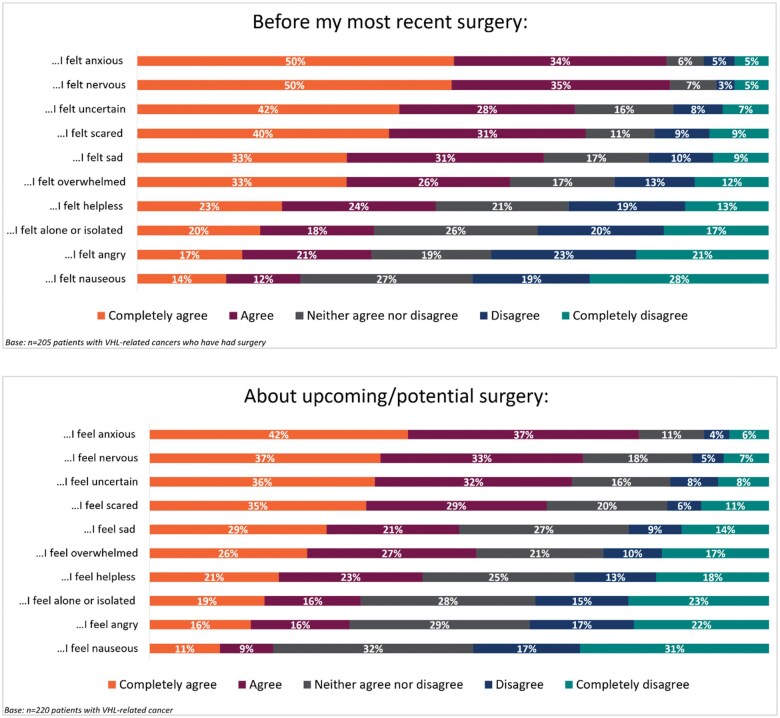
Patient’s expectations regarding their most recent surgery and any upcoming/potential surgery. VHL, Von Hippel-Lindau disease.

Overall, 205 (93.2%) patients had experienced at least one VHL-related surgery. Among these, 171 (77.7%) patients experienced multiple surgeries (median number of surgeries was 4.0). A total of 166 (75.5%) patients recorded data on their most recent surgery and the impact of the most recent surgery is provided below. Patients rated their experiences with surgery on a scale of 1 (greatly worsened) to 7 (greatly improved). Patients who had experienced at least one VHL-related surgery reported that their most recent surgery worsened (scored 1-3) their fatigue (*n* = 86, 51.8%), mental health (*n* = 85, 51.2%), and ability to go about daily life (*n* = 75, 45.2%) (Figure 1). Patients’ expectations for their most recent surgery and feelings about any upcoming/potential surgery is shown in Figure 2.

Approximately, 47.3% (n = 104) of patients selected “reduce the number of surgeries” as a top treatment goal, 46.4% (*n* = 102) selected “being able to go about life as usual,” 36.8% (*n* = 81) chose “delay the need for surgery,” and 33.2% (n = 73) chose “improved quality of life.” In all, 171 (77.7%) patients had 2 or more surgeries, 34 (15.5%) had 1 surgery, and 15 (6.8%) had never had surgery. Around 48.5% (*n* = 83) of patients who had 2 or more surgeries wanted to reduce their number of surgeries (44.1%, *n* = 15, for those who had had one surgery, 40.0%, *n* = 6, for those who had no previous surgeries), 44.4% (*n* = 76) of patients wanted to be able to go about life as usual (50% [*n* = 17] and 60.0% [*n* = 9] for 1 and no surgeries, respectively), and 37.4% (*n* = 76) wanted to delay the need for surgery (26.5% [*n* = 9] and 53.3% [*n* = 8] for 1 and no surgeries, respectively). Table 2 summarizes patient experience with surgery by number of surgeries.

**Table 2.** Experience with surgery by number of surgeries.

	Overall	0 surgeries	1 surgery	2+ surgeries
*n*, (%)	220 (100)	15 (6.8)	34 (15.5)	171 (77.7)
How many VHL-related surgeries have you ever had, median (interquartile range)	3.0 (2.0-7.0)	0.0 (0.0-0.0)	1.0 (1.0-1.0)	4.0 (2.0-8.0)
How many VHL-related surgeries have you ever had, n (%)				
0 surgeries	15 (6.8)	15 (100)	0 (0)	0 (0)
1 surgery	34 (15.5)	0 (0)	34 (100)	0 (0)
2+ surgeries	171 (77.7)	0 (0)	0 (0)	171 (100)
Top 3 goals for VHL-related cancer treatment, *n* (%)				
Reduce the number of surgeries	104 (47.3)	6 (40)	15 (44.1)	83 (48.5)
Being able to go about life as usual	102 (46.4)	9 (60)	17 (50)	76 (44.4)
Delay the need for surgery	81 (36.8)	8 (53.3)	9 (26.5)	64 (37.4)
Improved quality of life	73 (33.2)	2 (13.3)	8 (23.5)	63 (36.8)
Being able to plan for the future	65 (29.5)	7 (46.7)	13 (38.2)	45 (26.3)
Improve mental wellbeing	38 (17.3)	2 (13.3)	11 (32.4)	25 (14.6)
Achieving good balance between benefit of treatment and safety/side effects	37 (16.8)	3 (20)	4 (11.8)	30 (17.5)
Reduction in severity of symptoms	34 (15.5)	0 (0)	3 (8.8)	31 (18.1)
Reduce the need of hospitalization	30 (13.6)	3 (20)	5 (14.7)	22 (12.9)
Reduction in frequency of symptoms	28 (12.7)	2 (13.3)	2 (5.9)	24 (14)
Being able to go to work/school	27 (12.3)	2 (13.3)	5 (14.7)	20 (11.7)
Reduce number of medications I need to take	9 (4.1)	1 (6.7)	1 (2.9)	7 (4.1)
Other	2 (0.9)	0 (0)	0 (0)	2 (1.2)
I don’t have any specific treatment goals	7 (3.2)	0 (0)	2 (5.9)	5 (2.9)

Abbreviation: VHL, Von Hippel-Lindau syndrome.

Patients with VHL-related tumors had an average health state (EQ-5D-5L, US tariff) of 0.728. Patients who had experienced 2 or more surgeries had an average health state of 0.705, patients who had one surgery had an average health state of 0.803, and patients who had no surgeries had an average health state of 0.825.

**Figure 3.**
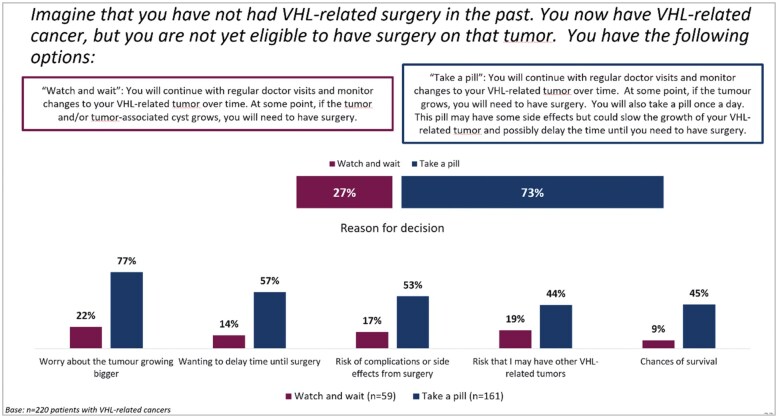
Patient preference scenario 1. VHL, Von Hippel-Lindau disease.

**Figure 4.**
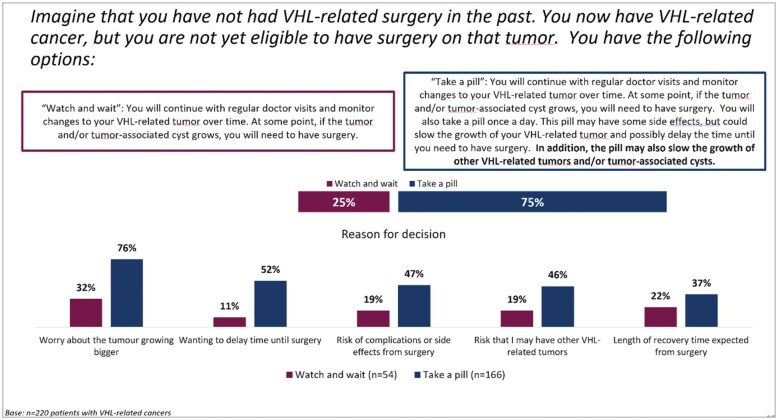
Patient preference scenario 2. VHL, Von Hippel-Lindau disease.

About 73.2% (*n* = 161) of patients indicated they would prefer to take a pill that would possibly delay the time until surgery, rather than watch and wait to see if the tumor would grow (Figure 3). When the scenario was modified to additionally include “**In addition, the pill may also slow the growth of other VHL-related tumors and/or tumor-associated cysts,**” it was even higher. Around 75.5% (*n* = 166) of patients indicated they would prefer to take a pill that would possibly delay the time until surgery, rather than watch and wait to see if the tumor would grow (Figure 4). The preference for a pill was affected by the number of previous surgeries. Specifically, of patients who have had no surgeries, 66.7% (*n* = 10) would take a pill; of patients who have had 1 surgery, 47.1% (*n* = 16) of patients would take a pill; and of patients who have had 2 or more surgeries, 78.9% (*n* = 135) would take a pill.

Reasons given for wanting to take a pill were “worry about the tumor getting bigger” (77.0%, *n* = 124), “wanting to delay time to surgery” (57.1%, *n* = 92), “risk of complications or side effect from surgery” (52.8%, *n* = 85), “chances of survival” (44.7%, n = 72), “risk that I might have other VHL-related tumors” (44.1%, n = 71), and “length of recovery time expected from surgery” (38.5%, *n* = 62). A vast majority of the patients would be willing to take a pill (strongly agree or agree) that could slow the growth of their VHL-related tumor and delay the time until surgery. Approximately, 97.5% (*n* = 157) of patients would take a pill if it increased their overall chances of survival. Around 95.6% (*n* = 154) of patients would take a pill, even if it had mild side effects, while 64.6% (*n* = 104) of patients would take a pill, even if it had moderate side effects. Roughly, 92.5% (*n* = 149) of patients would agree to take a pill if their doctor recommends it and 88.2% (*n* = 142) would take a pill if it was covered by their insurance. Full patient preference data are presented in Table 3.

**Table 3.** Patient preference.

	Overall	Watch and wait	Take a pill
Based on Scenario 1^a^, which option would you choose?, *n*, (%)	220 (100)	59 (26.8)	161 (73.2)
Which of the following factors influenced your decision to choose response for Scenario 1?, *n* (%)			
Worry about the tumor growing bigger	137 (62.3)	13 (22.0)	124 (77.0)
Wanting to delay time until surgery	100 (45.5)	8 (13.6)	92 (57.1)
Risk of complications or side effects from surgery	95 (43.2)	10 (16.9)	85 (52.8)
Risk that I may have other VHL-related tumors	82 (37.3)	11 (18.6)	71 (44.1)
Chances of survival	77 (35.0)	5 (8.5)	72 (44.7)
Length of recovery time expected from surgery	71 (32.3)	9 (15.3)	62 (38.5)
Symptoms experienced from the tumor	67 (30.5)	17 (28.8)	50 (31.1)
Effectiveness of the pill	63 (28.6)	17 (28.8)	46 (28.6)
Advice from my doctor	63 (28.6)	20 (33.9)	43 (26.7)
Possible side effects from taking the pill	57 (25.9)	30 (50.8)	27 (16.8)
Experience with other surgeries	50 (22.7)	11 (18.6)	39 (24.2)
Impact on my home, work, or school responsibilities	43 (19.5)	10 (16.9)	33 (20.5)
Effectiveness of surgery for VHL-related tumors	40 (18.2)	12 (20.3)	28 (17.4)
Risk that I may need dialysis	35 (15.9)	3 (5.1)	32 (19.9)
Costs of the pill (including insurance co-pays)	33 (15.0)	13 (22.0)	20 (12.4)
Advice from others (spouse, partner, family, caregiver, or friends)	30 (13.6)	7 (11.9)	23 (14.3)
Costs of the surgery (including insurance co-pays)	28 (12.7)	5 (8.5)	23 (14.3)
Want to avoid taking a pill	15 (6.8)	12 (20.3)	3 (1.9)
Burden of taking a daily pill	11 (5.0)	9 (15.3)	2 (1.2)
Other	6 (2.7)	5 (8.5)	1 (0.6)
**Based on Scenario 2** ^b^ **, which option would you choose?, ** *n* **, (%)**	220 (100)	54 (24.5)	166 (85.5)
Which of the following factors influenced your decision to choose response for Scenario 2?, n (%)			
Worry about the tumor growing bigger	143 (65)	17 (31.5)	126 (75.9)
Wanting to delay time until surgery	92 (41.8)	6 (11.1)	86 (51.8)
Risk of complications or side effects from surgery	88 (40)	10 (18.5)	78 (47.0)
Risk that I may have other VHL-related tumors	86 (39.1)	10 (18.5)	76 (45.8)
Length of recovery time expected from surgery	73 (33.2)	12 (22.2)	61 (36.7)
Effectiveness of the pill	69 (31.4)	19 (35.2)	50 (30.1)
Symptoms experienced from the tumor	68 (30.9)	14 (25.9)	54 (32.5)
Chances of survival	66 (30)	6 (11.1)	60 (36.1)
Advice from my doctor	63 (28.6)	17 (31.5)	46 (27.7)
Experience with other surgeries	53 (24.1)	9 (16.7)	44 (26.5)
Possible side effects from taking the pill	45 (20.5)	23 (42.6)	22 (13.3)
Effectiveness of surgery for VHL-related tumors	41 (18.6)	13 (24.1)	28 (16.9)
Impact on my home, work, or school responsibilities	39 (17.7)	6 (11.1)	33 (19.9)
Risk that I may need dialysis	34 (15.5)	4 (7.4)	30 (18.1)
Advice from others (spouse, partner, family, caregiver, or friends)	32 (14.5)	8 (14.8)	24 (14.5)
Costs of the surgery (including insurance co-pays)	27 (12.3)	5 (9.3)	22 (13.3)
Costs of the pill (including insurance co-pays)	26 (11.8)	9 (16.7)	17 (10.2)
Want to avoid taking a pill	17 (7.7)	16 (29.6)	1 (0.6)
Burden of taking a daily pill	15 (6.8)	14 (25.9)	1 (0.6)
Other	9 (4.1)	5 (9.3)	4 (2.4)

Abbreviation: VHL, Von Hippel-Lindau syndrome.

a **Scenario 1:** Imagine that you have not had VHL-related surgery in the past. You now have VHL-related cancer, but you are not yet eligible to have surgery on that tumor. You have the following options:.

**1. “Watch and wait”:** You will continue with regular doctor visits and monitor changes to your VHL-related tumor over time. At some point, if the tumor and/or tumor-associated cyst grows, you will need to have surgery.

**2. “Take a pill”:** You will continue with regular doctor visits and monitor changes to your VHL-related tumor over time. At some point, if the tumor grows, you will need to have surgery. You will also take a pill once a day. This pill may have some side effects but could slow the growth of your VHL-related tumor and possibly delay the time until you need to have surgery.

b **Scenario 2:** Imagine that you have not had VHL-related surgery in the past. You now have a VHL-related tumor, but you are not yet eligible to have surgery on that tumor. You have the following options:.

**1. “Watch and wait”:** You will continue with regular doctor visits and monitor changes to your VHL-related tumor over time. At some point, if the tumor and/or tumor-associated cyst grows, you will need to have surgery.

**2. “Take a pill”:** You will continue with regular doctor visits and monitor changes to your VHL-related tumor over time. At some point, if the tumor and/or tumor-associated cyst grows, you will need to have surgery. You will also take a pill once a day. This pill may have some side effects, but could slow the growth of your VHL-related tumor and possibly delay the time until you need to have surgery. In addition, the pill may also slow the growth of other VHL-related tumors and/or tumor-associated cysts.

## Discussion

The aim of this study was to examine the impact of VHL-related tumors on the lives of patients by assessing the burden of symptoms and current treatments. The study also explored patients’ preference for treatment that could alleviate the need for surgery versus waiting to see how the tumor may develop.

This study demonstrates that surgery presents a considerable burden upon the lives of patients with VHL disease. Patients with VHL disease develop multiple tumors in the brain, spine, retina, kidneys, and endocrine pancreas, among other organs. Surveillance for these tumors consists of annual imaging of the target organs. Craniotomies or spine surgeries may result in balance problems, dysesthesias, motor deficits, deficits in swallowing, and pain perception. Repeated partial nephrectomies may eventually compromise the renal function leading to chronic renal insufficiency renal failure, requiring hemodialysis, and/or transplantation.

The prospect of serial interventions negatively impacts the lives of patients,^11^ leading to a worsening in their fatigue, mental health, and ability to go about daily life. Patients of VHL have—due to their disease—a reoccurring psychological trigger and thus an ongoing impact on their quality of life. This study highlights this fact by showing that almost half of the VHL patients surveyed would prefer a treatment that reduces the number of surgeries, and over a third would prefer a treatment that delays the need for surgery. More specifically, nearly three quarters of patients would prefer to take a pill that might delay the need for surgery. In general, it can be said, that any treatment option that reduces the need for surgery would benefit this patient group.

The results of this study have also shown that most patients with VHL-related tumors have experienced surgery, many on more than one occasion. Even though, surgical management may successfully address VHL-related RCC—as the most frequent type of malignant tumor in VHL—it does not prevent new tumors from forming in the remaining, or contralateral, kidney.^4^ Studies indicate that a proportion of patients experience recurrence, necessitating additional surgeries. Over time these repeated surgical interventions can lead to renal insufficiency, potentially requiring dialysis or kidney transplantation.^4^

One of the top symptoms among patients with VHL-related tumors, as shown in this study, is fatigue, with surgery negatively affecting the energy a patient has in general. Altogether, there is a mental toll of an approaching surgery, with most patients agreeing that they felt nervous, anxious, and scared before their most recent surgery, as well as about unforeseen upcoming surgeries. Thus, reducing the need for surgical intervention was a top treatment goal, with about half of patients wanting to reduce their number of surgeries with a new treatment option.

Considering the reported impact that surgeries have on mental health, as discussed above, when offered the choice to either watch and wait to see how their tumor develops, or take a daily pill that might delay surgery, almost three quarters of patients would take the pill—even if it had mild/moderate side effects.

In light of the study’s findings on the impact of surgery on patients with VHL disease and their treatment preferences, potential avenues for future research may encompass the exploration of nonsurgical treatment options. This could involve the investigation of non-surgical modalities like targeted therapies, with the aim of alleviating tumor burden or delaying tumor growth in VHL disease. Furthermore, additional research efforts are warranted to deepen our understanding of the effects of VHL-related surgeries on patient reported outcomes, more specifically, mental health and quality of life.

The design of this study was cross-sectional, meaning the information captured from the patient survey is at a single point in time. Therefore, components of the study cannot be used to demonstrate cause and effect. However, the external validity (the ability of a study to be generalized to the real world) tends to be higher with non-interventional trials compared to clinical trials. Patient-reported outcomes tools utilized in the survey are not specifically validated in VHL. However, the quantitative survey was validated with patient piloting to ensure that the survey was methodologically rigorous in the collection of data. Patients were not recruited directly from a study physician, so VHL diagnosis was self-reported. In addition, another limitation of this study is the overrepresentation of female patients, which could be attributed to the survey’s sampling design. However, patients were recruited from a recognized patient advocacy group, VHL alliance, who have a goal of improving the quality of life and health outcomes for VHL patients.

## Data Availability

All data, that is, methodology, materials, data and data analysis, that support the findings of this survey are the intellectual property of Merck Sharp & Dohme LLC, a subsidiary of Merck & Co., Inc., Rahway, NJ, USA. The data underlying this article will be shared on reasonable request to Murali Sundaram of Merck & Co., Inc., Rahway, NJ, USA, murali.sundaram@merck.com.
